# Upper Limb Interactive Weightless Technology-Aided Intervention and Assessment Picks Out Motor Skills Improvement in Parkinson's Disease: A Pilot Study

**DOI:** 10.3389/fneur.2020.00040

**Published:** 2020-02-14

**Authors:** Cira Fundarò, Carlo Cavalieri, Gian Domenico Pinna, Anna Giardini, Francesca Mancini, Roberto Casale

**Affiliations:** ^1^Neurophysiopathology Unit, Istituti Clinici Scientifici Maugeri, IRCSS, Montescano, Italy; ^2^Neuromotory Rehabilitation Unit 1, Istituti Clinici Scientifici Maugeri, IRCSS, Montescano, Italy; ^3^Department of Biomedical Engineering, Istituti Clinici Scientifici Maugeri, IRCSS, Montescano, Italy; ^4^Psychology Unit, Istituti Clinici Scientifici Maugeri, IRCSS, Montescano, Italy; ^5^U. O. Neurologia—Stroke Unit e Laboratorio di Neuroscienze, Istituto Auxologico, IRCCS, Milan, Italy; ^6^OPUSMedica PC&R, Persons, Care and Research, Piacenza, Italy

**Keywords:** Parkinson's disease, upper limb rehabilitation, high technology rehabilitation, augmented feedback exercises, outcome measures

## Abstract

**Background:** In Parkinson's disease, reaching movements are conditioned by motor planning and execution deficiency. Recently, rehabilitation, aided by high technological devices, was employed for Parkinson's disease.

**Objective:** We aimed to (1) investigate the changes in the upper limb motor performances in a sample of a patient with Parkinson's disease after a weightless training, with a passive exoskeleton, in an augmented-feedback environment; (2) highlight differences by motor parameters (performance, speed, and movement accuracy) and by type of movement (simple or complex); and (3) evaluate movement improvements by UPDRS II–III.

**Methods:** Observational pilot study. Twenty right-handed patients with Parkinson's disease, Hohen and Yahr 2, Mini Mental State Examination ≥24 were evaluated. All patients underwent 5 day/week sessions for 4 weeks, 30 min for each arm; the training was performed with 12 exercises (single and multi-joints, horizontal and vertical movements). All the patients were assessed by UPDRS II–III and the evaluation tests provided by the device's software: a simple movement, the vertical capture, and a complex movement, the horizontal capture. For each test, we analyzed reached target percentage, movement execution time, and accuracy.

**Results:** After training, a significant improvement of accuracy and speed for simple movement on the dominant arm, of reached targets and speed for complex movement on both sides were shown. UPDRS II and III improved significantly after training.

**Conclusions:** In our study, a motor training aided by a high technological device improves motor parameters and highlights differences between the type of movement (simple or complex) and movement parameters (speed and accuracy) in a sample of patients with Parkinson's disease.

## Introduction

Parkinson's disease (PD) is a chronic progressive movement disorder, particularly affecting the reaching and grasping upper limb movements, which progressively become slow and difficult (1, 2), greatly impairing the activities of daily living (3, 4). Upper limb involvement in PD essentially regards deficiency in planning and in executing both voluntary simple and complex movements, as well as a lack in repeating motor poly-articular rapid sequences, attributed to dopaminergic pathway damage (5–7). This deficit produces slow and less precise reaching movements with a higher percentage of errors in movement accuracy as speed of action increases (8–10).

In the last few years, new models of PD rehabilitation were introduced, based on compensatory strategies, that are able to promote movements by bypassing the dopaminergic damage pathways (11). With these premises, upper limb rehabilitation, supported by a robotic or mechanic device, often in a virtual reality or augmented feedback environment, was proposed in order to execute intensive, repetitive, and task-oriented training in Parkinson's disease (11). These devices were originally employed in the rehabilitation of hemiplegia following brain vascular damage (12). Later, a similar training was subsequently first adopted for gait PD rehabilitation (13, 14).

At the best of our knowledge, few clinical studies in PD exist, which involve the adoption of a robotic or a mechanic device in order to improve upper limb mobility (15–17). Among these, exoskeleton devices aided by augmented feedback exercises, actually available for routine use, demonstrated successful results particularly in stroke rehabilitation (18, 19).

In addition, standardized outcome measures, in order to quantify rehabilitative training effects (3), are crucial in rehabilitation for Parkinson's disease as well and also when aided by robotic or mechanic devices (20). Precise and sophisticated movement analysis of motor performance achieved with instrumental rehabilitative devices, such as for instance kinematic analysis, was mostly carried out in the laboratory setting and are not easily adaptable for clinical routine (21, 22). For this purpose, the device used in our study was already used in patients with stroke to assess changes as outcome motor tasks after upper limb rehabilitation (23).

In this pilot study, we aimed to (1) investigate the changes in upper limb motor performances in a sample of a patients with PD after upper limb training with an exoskeleton device in an augmented feedback environment; (2) highlight differences by motor parameters (performance, speed, and movement accuracy) and type of movement (simple or complex); (3) evaluate ability and movement improvements by the Unified Parkinson Disease Rating Scale (UPDRS) II and III score.

## Methods

A sample of patients with PD, performing an in-patient rehabilitative training in the Neurorehabilitation and Neurophysiology Units of Montescano Medical Centre, ICS Maugeri, were enrolled in this pilot open study. The inclusion criteria were diagnosis of idiopathic Parkinson's disease according to the UK Brain Bank Criteria (24), bilateral disease symptoms [Hohen and Yahr (H&Y) stage 2] (25), and Mini Mental State Examination (MMSE) ≥ 24 (26). The exclusion criteria were severe dyskinesia or invalidating tremors, concomitant severe visual deficits, or neurological and/or orthopedic affections involving the upper limb, which could interfere with the use of the device.

The study was approved by our institutional review board and Central Ethical Committee (CEC) (approval number: CEC N.2042). All patients signed a consent form upon authorization to participate in the study and to access their medical records; in addition, data was managed anonymously. Patients continued their usual dopaminergic medications that remained unchanged during the duration of the study and for at least 4 weeks prior to its starting.

They were trained and tested during the “on” phase [after roughly 1.5 h from the last use of levodopa (LD)].

All exercises and evaluation tests were applied to the right and left arm for all patients.

Participants did not perform any type of rehabilitation in the 3 months before the study.

### Treatment Procedures

All patients underwent 5 day/week sessions of bilateral upper limb mechanic-aided treatment for 4 weeks, 30 min for each arm.

**Figure 1:**
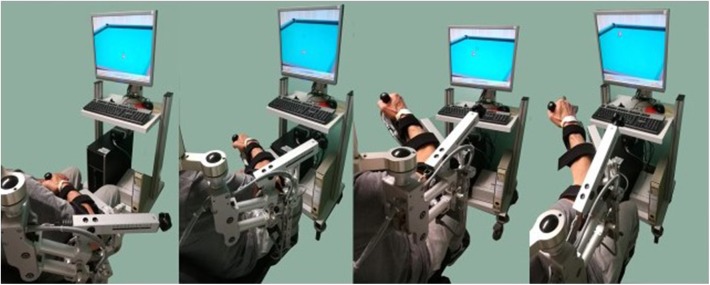
Armeo Spring®, patient position in the mechanical arm support and horizontal movement (HC) are represented. Patients sit on a non-slip chair; the device is linked to a screen, placed at 1.5 m in front of the patient, allowing for simultaneous reproduction of movements performed by the patient; a photographic sequential representation of horizontal capture movement is also displayed. The patient depicted in the figures has provided consent to publish his image.

The training was performed with the Armeo Spring® System (Hocoma, Zurich, Switzerland). Armeo Spring® is a mechanic exoskeleton device that reproduces the anatomical structure of the upper limb. The mechanical support is equipped with eight joints and with a handle with which the patient can execute a grab gesture; it is a gravity support instrument with a graduated spring system (Figure 1). The adjustment of the device was carried out according to the anthropometric features of the patient's limb; the easing of the weight of the arm and forearm was set through the spring system, adapting it to the strength and ability of the user. The device allows flexion/extension of the shoulder and elbow, abduction/adduction of the shoulder, protraction/retraction of the shoulder, prono-supination of the wrist, and grasping movements.

Armeo®'s software is provided with several goal-oriented training exercises in an augmented feedback environment, implemented with visual and acoustic stimuli and with a system that records the data in order to allow for further analysis.

An explanation and demonstration of the requested movement by a physiotherapist preceded the execution of the exercise, which was carried out without additional help.

**Figure 2:**
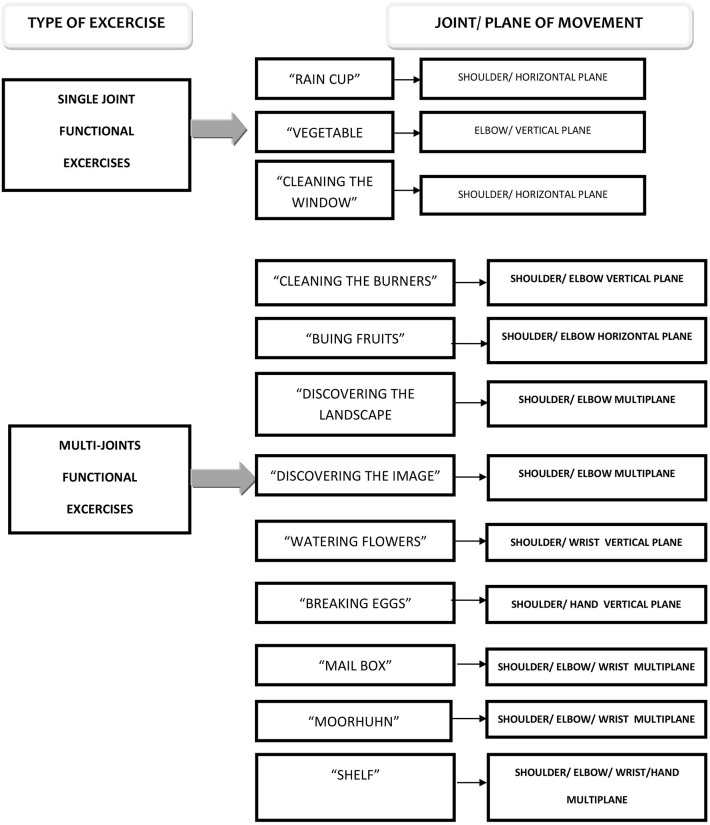
Exercises for training described by plane of movement, joints involved, and progressive complexity of the exercises.

Twelve functional goal-oriented exercises in an augmented feedback environment were employed with the involvement of different joints (single joint–three exercises—two for the shoulder and one for the elbow; multi-joints–nine exercises—four for the shoulder/elbow, one for the shoulder/wrist, one for the shoulder/grasping, one for the shoulder/elbow/wrist/grasping, and two for the shoulder/elbow/grasping), employing single movement, vertical or horizontal (seven exercises, four vertical, and three horizontal) plane or multi-plane motion (five exercises; Figure 2).

The exercise difficulty level during training, based on the number of targets to achieve, was selected from two (easy) to three (medium) according to this criterion: when 50% of the targets was reached, patients moved on to the next difficulty level, maintained until the end of the training.

Each functional exercise was selected with the same duration time of 2 min. Each patient carried out the same exercises in the same order of presentation from single to multi-joint exercises.

### Testing Procedures

The UPDRS II and III (27) administration and the evaluation tests provided by Armeo Spring®'s software (see below) were carried out by all the patients at T0, before the start of the training, and at T1, 1 day after the end of the training.

### Outcome Measures

The UPDRS II–III (27) (range, respectively: 0–52, 0–108) was used to evaluate the disease severity and disability. The same rater evaluated the patients at T0 and T1.

The motor performance measures were provided by the Armeo®'s software. These tests were vertical capture (VC) and horizontal capture (HC).

In the vertical capture test (VC), the patients have to reach a target (represented by a ladybird) by vertical movement. A simple movement with flexion–extension of the scapula–humerus joint and flexion–extension of the elbow is required to reach the target in the frontal plane.

In the horizontal capture test (HC), the patients must reach a target (billiard ball) by a horizontal movement, moving the arm in a transverse plane represented by a billiard ball. The required movement is complex, multi-articular, and composed of multi-stage sequential sub-movements (flexion–extension/abduction–adduction of the gleno-humeral joint, the protraction and retraction of the shoulder, and a repeated flexion–extension movement to reach the target; Figure 1).

For the evaluation test, a level 2 difficulty (scale 1–4, from very easy to difficult, equivalent to the number of targets to achieve) was chosen (23) for all tests both in T0 and T1, in order to ensure that the results of the evaluation were comparable over time. For level 2, 20 targets have to be reached in a time frame of 2 min.

Outcome measures for VC and HC are the number of the vertical and horizontal targets reached out of 20, expressed as percentage (%), i.e., VC and HC%; trajectory accuracy (a) for vertical and horizontal movements, respectively, measured as the ratio between the patient's hand trajectory in length and the ideal distance among the targets to reach, set by the device's software, where 1 is the best performance possible and where values higher than 1 indicate lower performance, i.e., VCa and HCa; the total time (t) of test execution for vertical and horizontal movements is expressed in seconds, i.e., VCt and HCt.

### Statistical Analysis

Descriptive statistics are given as mean ± SD (normally distributed data) or as median (lower quartile, upper quartile) (non-normally distributed data). Data from the Armeo Spring® device was analyzed by one-way ANOVA for repeated measures or the Wilcoxon signed-rank test, when appropriate. All tests were two-sided, and *p* < 0.05 was considered statistically significant. Analyses were performed using the SAS/STAT statistical package, release 9.2 (SAS Institute Inv., Cary, NC, USA).

## Results

Twenty right-handed subjects suffering from PD (five women and 15 men) were selected. The demographic and clinical characteristics of the patients are reported in Table 1.

**Table 1:** Description of the population and data results.

**Demographic and clinical characteristics of the patients**		**ANOVA results of the analysis of Armeo^®^ parameters of right and left arms as a function of time (post-training vs. baseline) during vertical (VC) and horizontal capture (HC) tests**					
		**Armeo Spring® parameters**	**Side treated**	**Baseline (T0)**	**Post-training (T1)**	**ΔT1–T0**	***P* (T1 vs. T0)**
NR	20	VC%	Right Left	100 (90, 100) 100 (95, 100)	100 (95, 100) 100 (97, 100)	0 (0, 5) 0 (0, 0)	0.20 0.53
Disease side onset	Left onset side 5 Right onset side 15	VCa	Right Left	1.27 ± 0.17 1.22 ± 0.06	1.20 ± 0.09 1.23 ± 0.10	−0.06 ± 0.16 0.02 ± 0.09	0.009 0.56
Gender	M15; F5	VCt	Right Left	63.8 ± 17.0 52.5 ± 12.4	52.6 ± 17.0 49.0 ± 9.4	−11.2 ± 15.8 −3.5 ± 11.2	0.006 0.19
UPDRS II	14.3 ± 5 (T0) 10.8 ± 4 (T1)	HC%^*^	Right Left	72 (61, 77) 75 (66, 83)	80 (77, 94) 83 (72, 88)	14 (0, 28) 6 (0, 11)	0.003 0.03
UPDRS III	29.1 ± 12.5 (T0) 22.3 ± 10.8 (T1)	HCa^*^	Right Left	1.44 ± 0.45 1.59 ± 0.24	1.60 ± 0.29 1.61 ± 0.27	0.15 ± 0.49 0.03 ± 0.28	0.20 0.70
Disease duration	4.9 ± 2.2	HCt^*^	Right Left	89.7 ± 21.6 85.1 ± 20.8	76.6 ± 15.1 76.4 ± 19.8	−13.1 ± 14.9 −8.7 ± 12.7	0.002 0.01
LED	664.5 ± 240.9						

*The sample's clinical characteristics (gender, disease onset side, disease duration expressed in years, UPDRS II–III, levodopa daily total dosage LED expressed in mg) are reported; all the results are reported: data are expressed as mean ± SD (normally distributed data), or as median (lower quartile, upper quartile; non-normally distributed data). VC%, % of targets reached (ordinal number); VCa, trajectory accuracy (ordinal number); VCt, time execution test in seconds (s); HC%, % of targets reached (ordinal number); HCa, trajectory accuracy (ordinal number); HCt, time execution test in seconds (s)*.

* *Analysis was performed in 18 subjects owing to failure to execute the baseline evaluation in two subjects*.

All the patients at the time were under pharmacological therapy: all subjects under LD, for 10 subjects in association with dopamine agonists (DA) (five patients with pramipexole, three with rotigotine, two with ropinirole); 10 patients were also under rasagiline therapy. Therapy is expressed as the total daily levodopa equivalent dose (LED) (see Table 1) (28).

Regarding the UPDRS II–III score, significant improvement is observed from baseline to post-training, respectively, with a reduction from 14.3 ± 5 to 10.8 ± 4 (*p* < 0.0001) and 29.1 ± 12.5 to 22.3 ± 10.8 (*p* < 0.0001; see Table 1).

**Figure 3:**
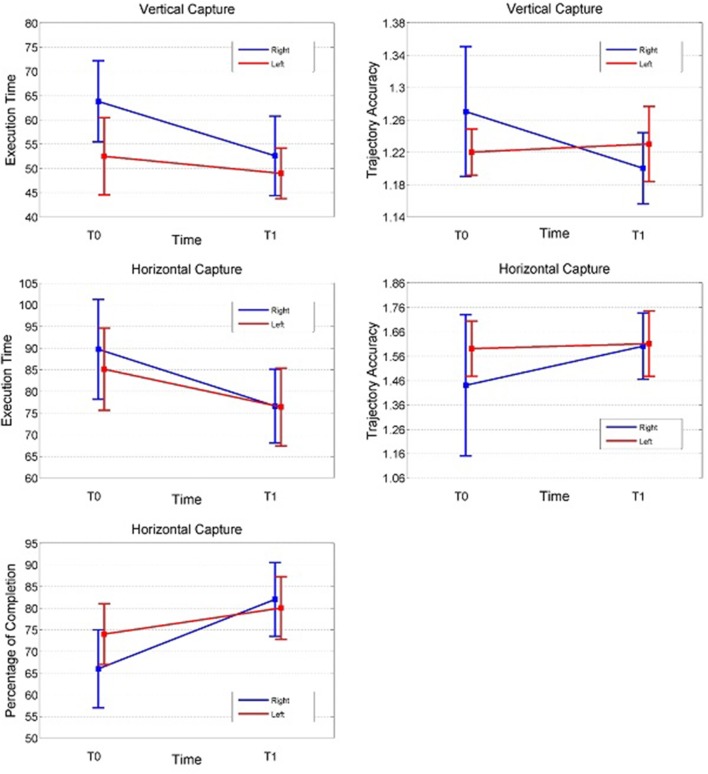
Result representation (VCa, VCt at T0–T1, HC%, HCa, HCt at T0–T1) in both sides; the right side is represented with a circle and the left side with a square; in ordinate percentage of success completion expressed in %, trajectory accuracy expressed as ordinal number, total time execution expressed as seconds, and in abscissa the baseline and final time evaluation; VC% percentage of success completion is not represented (100% for the right and left sides at T0 and T1).

Armeo Spring® evaluation parameters and ANOVA results for vertical capture (VC) and horizontal capture (HC) tests performed at baseline and after training are reported in Table 1 and Figure 3.

The following is a detailed description of each test.

### Vertical Capture Baseline Measurements

The percentage of targets reached at baseline was close to 100% in each side. There were no significant differences regarding trajectory accuracy between the right and left sides. The execution time (VCt) was higher on the right side (*p* = 0.006).

### Vertical Capture Post-training Measurements

The percentage of targets reached close to 100% at baseline and did not significantly increase at the end of the training program.

A significant improvement in trajectory accuracy (VCa) and execution time test (VCt) on the right side (*p* = 0.009 and *p* = 0.006, respectively) was noted, while no significant differences were observed on the left side.

### Horizontal Capture Baseline Measurements

We clarify that analysis of the data from the HC test was performed in 18 subjects only, due to failure to execute baseline evaluation in two subjects; these patients, however, were able to fulfill the test at post-training evaluation.

All baseline measurements presented no significant differences between the right and left sides (*p* ≥ 0.08 for all comparisons).

The percentage of targets reached (HC%) at baseline was lower than that observed in the VC tests.

Overall, the HC results were characterized by a larger inter- and intra-subject variability compared to those from the VC tests, as evidenced by the magnitude of standard deviations.

### Horizontal Capture Post-training Measurements

The percentage of targets reached (HC%) improved significantly after training on both sides (right *p* = 0.003, left *p* = 0.03). A significant bilateral improvement was also observed in the execution time test (HCt) (right *p* = 0.002, left *p* = 0.01). No significant change was observed in trajectory accuracy after training.

## Discussion

In our study, motor training aided by an upper limb mechanic exoskeleton in an augmented feedback environment improves motor parameters (performance, speed, and accuracy) and highlights the differences between the type of movement (simple or complex) and movement parameters (speed and accuracy) in a sample of patients with PD.

In detail, at baseline, motor performances in simple movements were close to 100%, while in complex movements (HC test), the percentage of targets reached was clearly lower than that observed in the simple movements (VC test); the execution time in VC was higher on the right side. After training, a significant improvement in accuracy and speed for simple movement (VC) on the dominant arm of reached targets and speed for complex movement (HC) were bilaterally shown; in complex movements, accuracy did not show significant improvements after training.

### Training Efficacy

Generally, regarding these findings, our data show an overall positive training effect in patients with PD as expressed in the literature (29).

This motor improvement is in agreement with the existing literature suggesting the involvement of complex and multiple motor learning mechanisms (30, 31), enhanced by numerous systems such as visual and acoustic cues or feedback stimuli (32, 33), repetition of focused tasks (34), reinforcement of attention skills (35), and a process arguably similar to action observation movements (36). In our opinion, many of these mechanisms were involved in mechanic or robotic rehabilitation with augmented feedback stimuli.

### Complex vs. Simple Movements

In a more in-depth analysis of each specific evaluation test, our data show several differences in movement characteristics related to the type of movement requested.

In the baseline vertical capture test, where a simple movement of the scapula humeral articulation is requested, all patients completed the task easily, as a rapid change in motor strategy was not needed (37). On the contrary, at baseline in the horizontal capture test movement, where a fast and sequential multi articular movement is necessary to reach the target, our data showed increased difficulty for patients with PD. Nevertheless, this measure also improved significantly after training.

After training, in the horizontal and vertical capture tests, an improvement in movement speed was observed, while an enhancement of trajectory accuracy was highlighted only in the simple movement of the vertical capture test.

In PD, deficiency in dopaminergic pathways produces slow and less precise reaching movements (38) with a higher percentage of errors as speed of action increases (8, 9). Various interpretations are currently under debate regarding how this deficit may impair movement by employing inadequate motor programs like incorrect muscular activation and impairment in motor learning (30).

It is well-known that patients with PD are able to complete tasks with adequate speed, at the cost of less accurate movements (10); in particular, it appears that this inability is more evident in complex multi articular efforts (8, 9). In other words, patients with PD were able to learn new motor strategies (30, 33, 39) and implement speed movements especially when supported by external stimuli during training (40), although accuracy of complex movements was not improved (8, 9, 38).

### Device as Assessment Tool

In addition, the possibility to measure the accuracy and time execution of movement is particularly relevant in Parkinson's disease, where these aspects represent the core of motor deficit and disability.

Clinical evaluation of motor performances after rehabilitative training is crucial for the possibility to quantify motor learning effect and need specific and objective instruments. In fact, the only use of a clinical scale—UPDRS—as a rehabilitative measure as well shows relevant limits; in fact, UPDRS, in itself, is not able to show specific differences regarding movement characteristics after rehabilitation. As a consequence, in order to better and more objectively describe movement improvement, other measure systems associated with UPDRS are often used in previous studies, i.e., kinematic analysis of movement (41, 42) or quantitative digitography for finger tapping testing (43) or the wearable motion capture system (44) were used.

In this direction, technologies may also be applied in order to assess bradykinesia in PD (45).

### Movement Side Differences in Parkinson's Disease

To conclude, in our results, the Armeo®'s evaluation software appears to underline certain differences in movement between disease sides: in vertical capture time execution, the upper right limb resulted mostly compromised at baseline (46, 47). The literature shows that the onset side in Parkinson's disease is the most compromised through all the patient's life (48) and the onset predominant side in PD patients is perhaps correlated to handedness (49, 50); in line with this account, in our right-handed subjects, most (15 subjects) were right-side onset. The pathophysiology background of the asymmetric motor impact in PD is, however, currently unsolved (51).

### Limits

Finally, some limits in our study may be stressed: first, being a pilot study on daily care clinical data, no control group is available; moreover, the small sample makes these results also preliminary and require further analysis, particularly for reproducibility. Starting from these considerations, future studies properly sized, controlled, or compared to another type of training and with a long-term follow-up evaluation would be of interest.

## Data Availability Statement

The datasets generated for this study are available on request to the corresponding author.

## Ethics Statement

The studies involving human participants were reviewed and approved by ICS Maugeri Institutional Review Board and Central Ethical Committee (CEC) (approval number: CEC N.2042). The patients/participants provided their written informed consent to participate in this study.

## Author Contributions

All authors read and approved the final manuscript. CF was responsible for the conception, organization, and execution of the study. She also assisted with developing the design and review of the statistical analysis. Finally, she assisted in the preparation, review, and critique of the manuscript. CC helped to organize and execute the study. He also assisted with the statistical analysis and review of the manuscript. GP assisted with the development of the statistical analysis. AG was involved with the conception of the study. She also assisted with the review of the manuscript. FM assisted with the conception of the study and the review of the manuscript. RC was involved with the conception of the study protocol and with the review of the manuscript.

### Conflict of Interest

The authors declare that the research was conducted in the absence of any commercial or financial relationships that could be construed as a potential conflict of interest.

## Abbreviations

PD: Parkinson's disease
H&Y: Hoehn and Yahr
MMSE: Mini Mental State Examination
VC: vertical capture
VCt: vertical capture total time execution
VCa: vertical capture accuracy
VC%: vertical capture percentage
HC: horizontal capture
HCt: horizontal capture total time execution
HCa: horizontal capture trajectory accuracy
HC%: horizontal capture percentage
UPDRS: Unified Parkinson's Disease Rating Scale
UPDRS II and III: Unified Parkinson's Disease Rating Scale sections II and III
VR: virtual reality
LD: levodopa
DA: dopamine agonist
LED: levodopa total daily dose.
